# BAGLS-VF: a comprehensive dataset for glottal area and vocal fold segmentations

**DOI:** 10.1186/s13104-026-07972-7

**Published:** 2026-08-17

**Authors:** Sina Razi, Andreas M. Kist

**Affiliations:** Department Artificial Intelligence in Biomedical Engineering, Friedrich-Alexander-Universität Erlangen-Nürnberg, Erlangen, Germany

**Keywords:** Laryngeal endoscopy, Dataset, BAGLS, Vocal folds, High-speed videoendoscopy, Anatomy, Voice, Speech, Dysphonia, Quantitative laryngology

## Abstract

**Objective:**

Datasets for automatic analysis of laryngeal endoscopic images are scarce, particularly those providing detailed anatomical annotations beyond the glottal area. The BAGLS dataset previously introduced a large collection of laryngoscopic images with expert annotations of the glottal area. To facilitate research on more detailed anatomical modeling and segmentation tasks, we introduce BAGLS-VF, an extension of the BAGLS dataset that includes additional annotations of the left and right vocal folds.

**Data description:**

BAGLS-VF contains 59,250 videolaryngoscopic frames with pixel-level annotations of three anatomical structures: glottal area, left vocal fold, and right vocal fold. The dataset is derived from the original BAGLS dataset and was semi-automatically annotated by a trained rater using a standardized annotation protocol. Images originate from high-speed videolaryngoscopic recordings of subjects undergoing clinical examination and cover a wide range of anatomical variability and imaging conditions. In line with the original BAGLS dataset, the images are predominantly grayscale (approximately 97% of the underlying recordings) with a small proportion of RGB recordings (approximately 3%); the segmentation masks are provided as RGB-encoded label images in which the three anatomical regions are stored as separate colour channels. BAGLS-VF is intended to support research in medical image segmentation, laryngeal biomechanics, and computer-assisted diagnosis.

## Objective

Machine learning methods have increasingly been applied to the analysis of laryngeal endoscopy and high-speed videoendoscopy data [1, 2]. However, progress in this field is limited by the availability of annotated datasets containing detailed anatomical structures. While several datasets provide annotations of the glottal area, few datasets contain additional annotations of the vocal folds themselves, which are necessary for studying vocal fold motion, symmetry, and structural abnormalities [1, 3, 4].

The BAGLS dataset [5] was previously introduced as a large collection of videolaryngoscopic images with expert annotations of the glottal area. While this dataset enabled a variety of segmentation and detection tasks, it did not include annotations for the left and right vocal folds separately.

To address this limitation, we generated BAGLS-VF, an extended dataset that supplements the original BAGLS images with additional pixel-wise annotations of the vocal folds. These annotations allow the separation of three anatomical structures:

- Glottal area.
- Left vocal fold.
- Right vocal fold.

The dataset aims to support research in semantic segmentation of laryngeal structures, the automated analysis of vocal fold motion, for example, in phonovibro- or laryngovibrograms [6, 7], machine learning approaches for voice disorder assessment, and the development of computer-assisted diagnostic tools [8]. By making these annotations publicly available, BAGLS-VF aims to increase reproducibility and support benchmarking of segmentation algorithms for laryngeal image analysis.

## Data description

The BAGLS-VF dataset consists of videolaryngoscopic frames derived from the BAGLS dataset. Each image is accompanied by a segmentation mask containing annotations of the glottal area and both vocal folds. Images were obtained from high-speed videolaryngoscopic examinations recorded during routine clinical assessments. The recordings were extracted into individual frames, resulting in a total of 59,250 images that are separated into a training set (55,750 images) and a test set (3,500 images). The BAGLS-VF dataset is available as two zip files for the training and test set, respectively (Table 1). The images are taken during phonation and show the vibrating vocal folds with the opening and closing of the glottal area.

Each image is accompanied by a segmentation mask with three anatomical labels, namely glottal area, left vocal fold, and right vocal fold. Each pixel is assigned a unique class, and the remaining pixels correspond to the background. Masks are stored as labelled images where pixel values encode the respective anatomical class. Example images and corresponding annotations are available in the repository.

Annotations were created using a semi-automatic segmentation workflow carried out by a single trained annotator (S.R.), who was trained by a medical domain expert (A.M.K.) and followed a standardized annotation protocol. In brief, the protocol entailed that (i) the glottal area was adopted unchanged from the original BAGLS ground-truth masks [5]; (ii) the left and right vocal folds were delineated so that the medial boundary of each fold coincides as good as possible with the corresponding edge of the glottal area, the lateral boundary follows the visible transition between the vocal fold and the surrounding laryngeal tissue, and the anterior and posterior extents are limited to the region in which the fold is clearly discernible; and (iii) ambiguous frames were resolved conservatively by annotating only the clearly identifiable portion of each fold.

For the vocal fold boundaries, SAM-2 [9] was used to obtain initial mask proposals. For each frame, the annotator placed a small number of positive point prompts (typically one to three) on the interior of each vocal fold and, where necessary, negative point prompts on the adjacent glottal area or background to suppress leakage across the anatomical boundary. The resulting proposals were then reviewed and, where required, corrected manually using the pipra tool [5] (https://github.com/anki-xyz/pipra).

All 59,250 frames were processed and manually reviewed by the annotator. During this review, each mask was assigned to one of three categories. A first group of 31,576 masks was considered acceptable after the SAM-2 step and required no or only negligible correction. Within this group, a custom-trained U-Net (see below) was subsequently applied to 12,007 masks to refine small imperfections along the vocal fold boundaries; the U-Net outputs were again visually inspected by the annotator and accepted only where they improved the mask, so that these 12,007 masks remain a subset of the 31,576 acceptable masks rather than an additional group. A second group of 20,714 masks showed significant inaccuracies, such as blurred boundaries, considerable anatomical misrepresentation, or extensive noise; these masks were corrected individually and manually using the pipra tool [5]. The remaining 6,960 masks required minor manual adjustments that went beyond the acceptable category but did not reach the level of significant inaccuracy; these were likewise refined manually using pipra. Every mask in the released dataset was manually reviewed by the annotator before inclusion.

The custom-trained U-Net (based on [10]) used for refinement was a standard encoder–decoder segmentation network trained on the subset of masks that had already been accepted, with the laryngoscopic image as input and the three-class mask (glottal area, left vocal fold, right vocal fold) as target. It was used exclusively as an assistive post-processing step to smooth small boundary imperfections and never as an unsupervised source. As described above, all of its outputs were manually inspected and were retained only where a human annotator confirmed an improvement.

The dataset repository (Table 1) contains the following files:

- Images: PNG files from the BAGLS dataset. Training images are provided cropped to 256 × 256 px, whereas test images are provided at their original resolution (see below).
- Segmentation masks: PNG files containing pixel-wise labels encoded as RGB (R = glottal area, G = right vocal fold, B = left vocal fold) corresponding to the associated image file. RGB here denotes the red–green–blue colour encoding of the mask channels.

Each image file is associated with a segmentation mask through a shared filename in two separate folders, for example 0.png for the laryngoscopic image and mask_0.png for the associated mask.

The training images were cropped to a uniform 256 × 256 px resolution to provide a ready-to-use, memory-efficient training set that can be loaded in fixed-size batches by common deep learning frameworks without further preprocessing, following common practice for the original BAGLS training pipeline [5]. The test images were kept at their original resolution so that benchmark scores reflect performance on unmodified clinical images and are not biased by a specific cropping or resizing scheme. This is aligned with the original BAGLS convention of reporting test scores on the native image geometry. Users who require a fixed input size for inference can apply their own resizing or padding to the test images while retaining full control over the preprocessing, which we consider important for fair and reproducible benchmarking.

BAGLS-VF can support a variety of research tasks, including the semantic segmentation of laryngeal structures, benchmarking of medical image segmentation algorithms, development of machine learning models for vocal fold analysis, and the simulation and modeling of vocal fold biomechanics. Because the dataset provides annotations for both vocal folds separately, it also enables studies of left–right symmetry and of the spatial configuration of the vocal folds.

## Limitations

Several limitations of the dataset should be considered. First, annotations were generated semi-automatically by a single rater and may therefore contain an annotator bias. Although the annotator followed a standardized protocol, segmentation of anatomical boundaries in laryngoscopic images can be ambiguous in some cases, and a formal inter-rater analysis of the vocal fold annotations on this dataset was not performed. As a point of reference, a previous study on the same underlying BAGLS image data, but restricted to glottal area segmentation and without SAM-2 assistance, reported an inter-rater agreement of approximately 0.7 IoU [11], indicating that laryngeal segmentation tasks in general are subject to limited inter-rater reliability. We note that this value was obtained for a different segmentation target and workflow and is therefore only an approximate indication rather than a direct measure of the reliability of the vocal fold annotations released here; a dedicated inter-rater study on a representative subset of BAGLS-VF is a valuable direction for future work. Second, the dataset is derived from videolaryngoscopic images acquired under clinical conditions, which may result in variability in image quality, illumination, and viewing angle. Third, the dataset represents individual static frames rather than full, temporally contiguous video sequences. While the separate annotation of both vocal folds enables the study of left-right symmetry and of the spatial configuration of the folds, the frame-based organization limits the direct temporal analysis of vocal fold vibration dynamics; studies requiring continuous motion trajectories would need to complement BAGLS-VF with the corresponding raw recordings of the original BAGLS dataset.

**Table 1:** Overview of BAGLS-VF

Label	Name of data file/data set	File types (file extensions)	Data repository and identifier (DOI or accession number)
Figure	Figure – Dataset Overview	.png	Zenodo (10.5281/zenodo.19593658) [12]
Data file 1	BAGLS-VF training	.zip – contains images and masks as.png	Zenodo (10.5281/zenodo.19593658) [12]
Data file 2	BAGLS-VF test	.zip – contains images and masks as.png	Zenodo (10.5281/zenodo.19593658) [12]

## Data Availability

The BAGLS-VF dataset is available on Zenodo, similar to the original dataset. We provide both the training and the test set under the same identifier at 10.5281/zenodo.19593658 [12].

## Abbreviations

BAGLS: Benchmark for Automatic Glottis Segmentation
BAGLS-VF: BAGLS with vocal fold annotations
IoU: Intersection over Union
PNG: Portable Network Graphics
RGB: Red, Green, Blue (colour channel encoding)
SAM-2: Segment Anything Model, version 2
U-Net: convolutional encoder–decoder network for image segmentation
